# Case Report: A modified approach to converting ventriculoperitoneal shunt to ventriculoatrial shunt due to recurrent encapsulation of the peritoneal catheter

**DOI:** 10.3389/fsurg.2025.1516115

**Published:** 2025-02-25

**Authors:** YunSen Zhang, YuanHong Ge, Yong Liu, Yue Zhang, RongHua Xu, Xuejun Xu

**Affiliations:** ^1^ Department of Neurosurgery, Chengdu Second People’s Hospital, Chengdu, Sichuan, China; ^2^ School of Clinical Medicine, Chengdu Medical College, Chengdu, Sichuan, China

**Keywords:** hydrocephalus, ventriculoperitoneal, ventriculoatrial, rapid exchange technique, case report

## Abstract

**Background:**

Hydrocephalus is a condition characterized by the accumulation of cerebrospinal fluid (CSF) in the ventricular system due to various causes, including excessive CSF production, impaired circulation, or absorption dysfunction. This condition is often accompanied by ventricular enlargement, compression of brain parenchyma, and increased intracranial pressure. Ventriculoperitoneal (VP) shunting is the first-line treatment for hydrocephalus; however, when the peritoneal catheter becomes obstructed due to encapsulation, the procedure may need to be converted to a ventriculoatrial (VA) shunt, which serves as a second-line treatment. Here, we present a case that demonstrates a rapid, simple, and minimally invasive technique for converting a VP shunt to a VA shunt. This approach eliminates the need to expose the retroauricular valve or disconnect the valve from the catheter, significantly reducing operative time and minimizing trauma.

**Case presentation:**

A 61-year-old male patient presented with typical clinical features of hydrocephalus, including urinary dysfunction, gait instability, and gradually worsening cognitive decline over the course of a year, as well as corresponding imaging findings. The patient subsequently underwent a VP shunt procedure. However, within six months postoperatively, the patient experienced four episodes of shunt dysfunction due to omental encapsulation of the peritoneal catheter, leading to catheter obstruction and worsening hydrocephalus. During the first three episodes, the shunt catheter was released from omental encapsulation through laparoscopic surgery, providing temporary relief of hydrocephalus after each procedure. Following the fourth episode of peritoneal shunt dysfunction, we employed a rapid exchange technique to relocate the peritoneal catheter to the superior vena cava while preserving the ventricular catheter and shunt valve. Postoperatively, the patient's hydrocephalus-related symptoms gradually improved. At the three-month follow-up, the patient's hydrocephalus showed significant improvement, and he had returned to independent daily living.

**Conclusion:**

The rapid exchange technique is a fast, simple, and minimally invasive method for converting a VP shunt to a VA shunt, offering significant benefits in clinical practice.

## Background

VP shunting is the first-line neurosurgical treatment for hydrocephalus (1, 2). However, it is sometimes necessary to modify the procedure to a VA shunt—also known as ventriculo-superior vena cava or atrial shunting—a second-line treatment, due to recurrent occlusion of the peritoneal end of the shunt catheter (3, 4). Traditionally, VA shunting requires exposing the internal jugular vein and making an incision to directly insert the drainage catheter (5, 6). This method, however, is time-consuming, more invasive, and carries higher risks. Some studies have reported a minimally invasive (percutaneous) technique for placing the atrial end of the drainage catheter into the superior vena cava or atrium (7, 8). This approach involves using an internal jugular vein catheterization puncture kit and applying the Seldinger technique to insert a “J”-shaped guidewire. The atrial end of the drainage catheter is then advanced over the guidewire into the superior vena cava or atrium, a method hereafter referred to as the “coaxial technique” to differentiate it from the technique described in this study. The placement of the ventricular catheter, the positioning of the retroauricular valve, and the connection of both ends of the catheter are performed in the same manner as in the classic VP shunt procedure.

When converting from VP to VAshunting, either the traditional method or the coaxial technique can be used. While the coaxial technique offers certain advantages over the traditional approach, it requires an additional incision at the retroauricular valve site to expose the valve, disconnect it from the peritoneal catheter, and, after placing the atrial catheter into the superior vena cava or atrium, remove the guidewire from the other end of the drainage catheter before reconnecting the valve.

This study introduces a fast, simple, and minimally invasive approach, which we have termed the “rapid exchange technique.” This method eliminates the need to expose the retroauricular valve or disconnect it from the catheter, significantly reducing surgery time and further minimizing trauma.

## Case presentation

**Figure 1:**
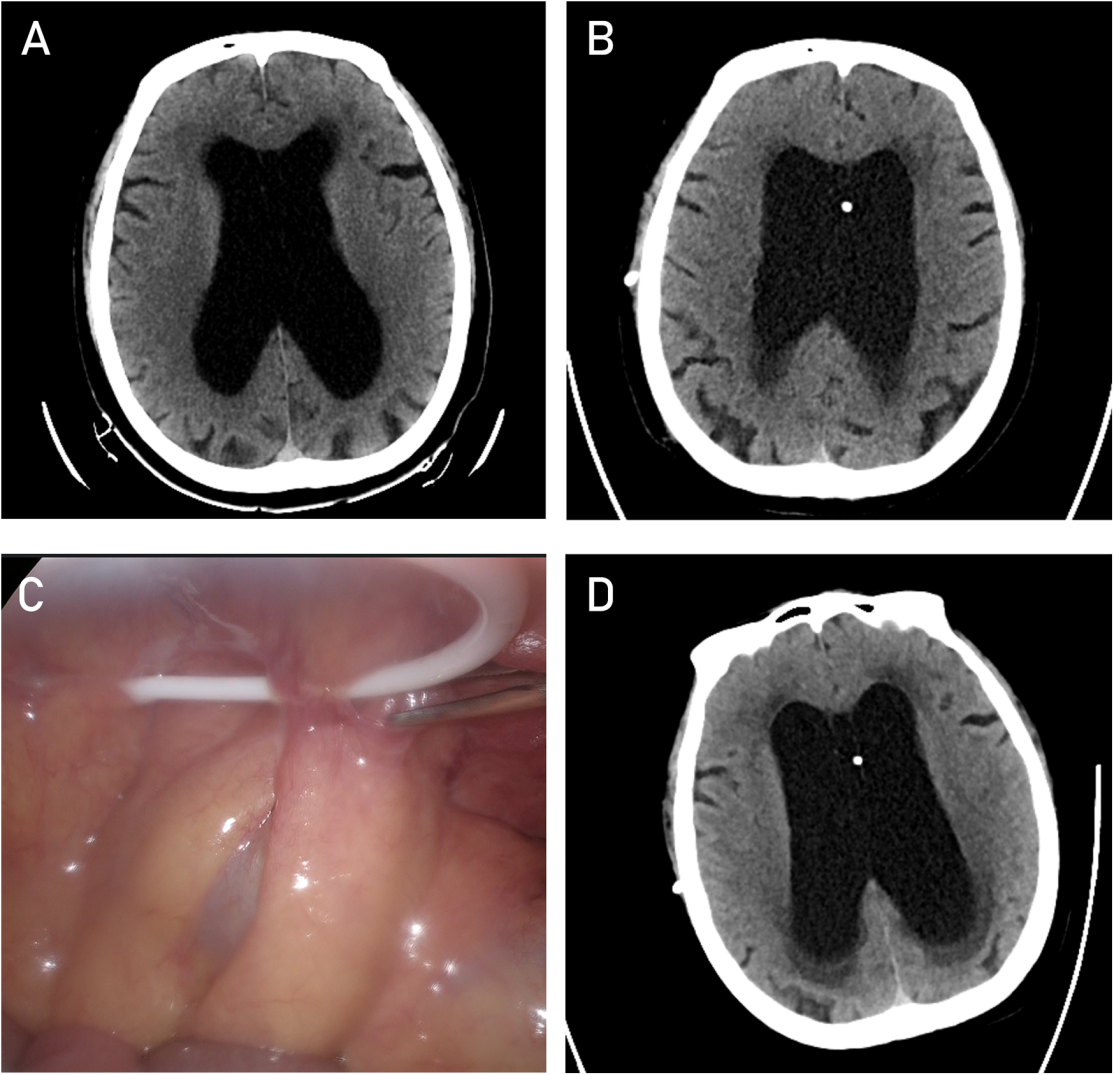
CT image of the lateral ventricles at the time of hydrocephalus diagnosis. **(A)** CT image of the lateral ventricles after the first VP shunt procedure. **(B)** Laparoscopic image showing the encapsulation of the peritoneal catheter. **(C)** CT image of the lateral ventricles after encapsulation of the peritoneal catheter **(D)**.

**Figure 2:**
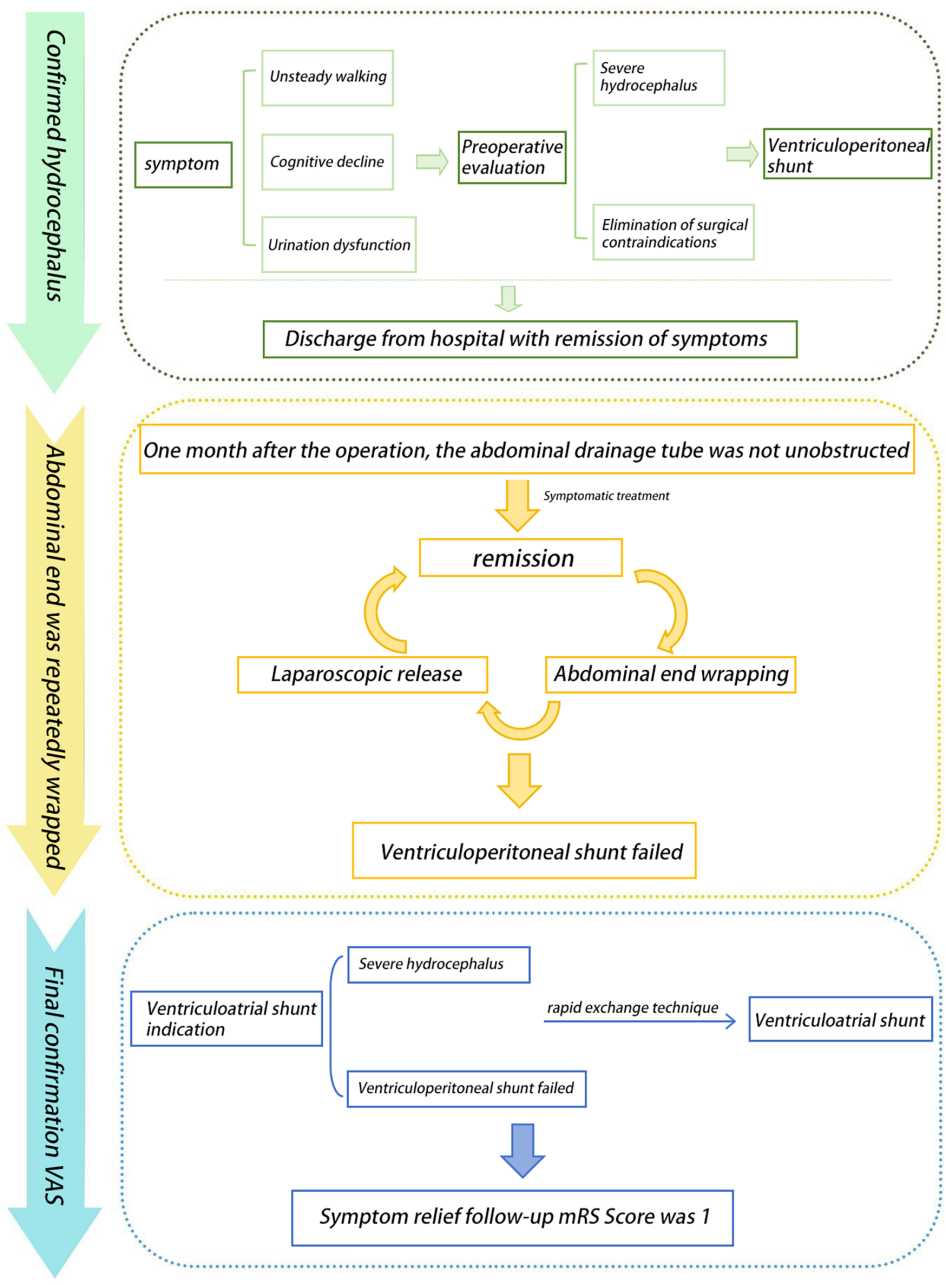
The complete timeline of the patient's disease progression from the confirmed diagnosis of hydrocephalus to VP treatment, and subsequently to conversion to VA shunting.

A 61-year-old male patient presented with typical clinical features of hydrocephalus, including urinary dysfunction, gait instability, and progressively worsening cognitive decline over the course of one year, along with corresponding radiological findings (Figure 1A). The patient subsequently underwent a VP shunt procedure (Figure 1B). However, within six months postoperatively, he experienced four episodes of shunt malfunction due to the peritoneal end of the shunt catheter becoming encased by the omentum (Figure 1C), resulting in obstructed drainage and worsening hydrocephalus (Figure 1D). After each obstruction, we performed imaging examinations and aspirated cerebrospinal fluid from the shunt valve for analysis. Considering the patient's symptoms, physical examination findings, and a comprehensive discussion among specialists in neurosurgery, radiology, and abdominal surgery, infection was ultimately ruled out. During the first three episodes, the shunt catheter was freed from the omental encasement through laparoscopic surgery, providing temporary relief of hydrocephalus after each procedure. During laparoscopic adhesiolysis, we collected CSF from the distal end of the shunt catheter and excised a segment of the catheter for examination, further ruling out infection. Following the fourth episode of peritoneal shunt malfunction, a decision was made to relocate the peritoneal end of the shunt to the superior vena cava while retaining the ventricular catheter and shunt valve. The clinical course is illustrated in the flowchart (Figure 2).

**Figure 3:**
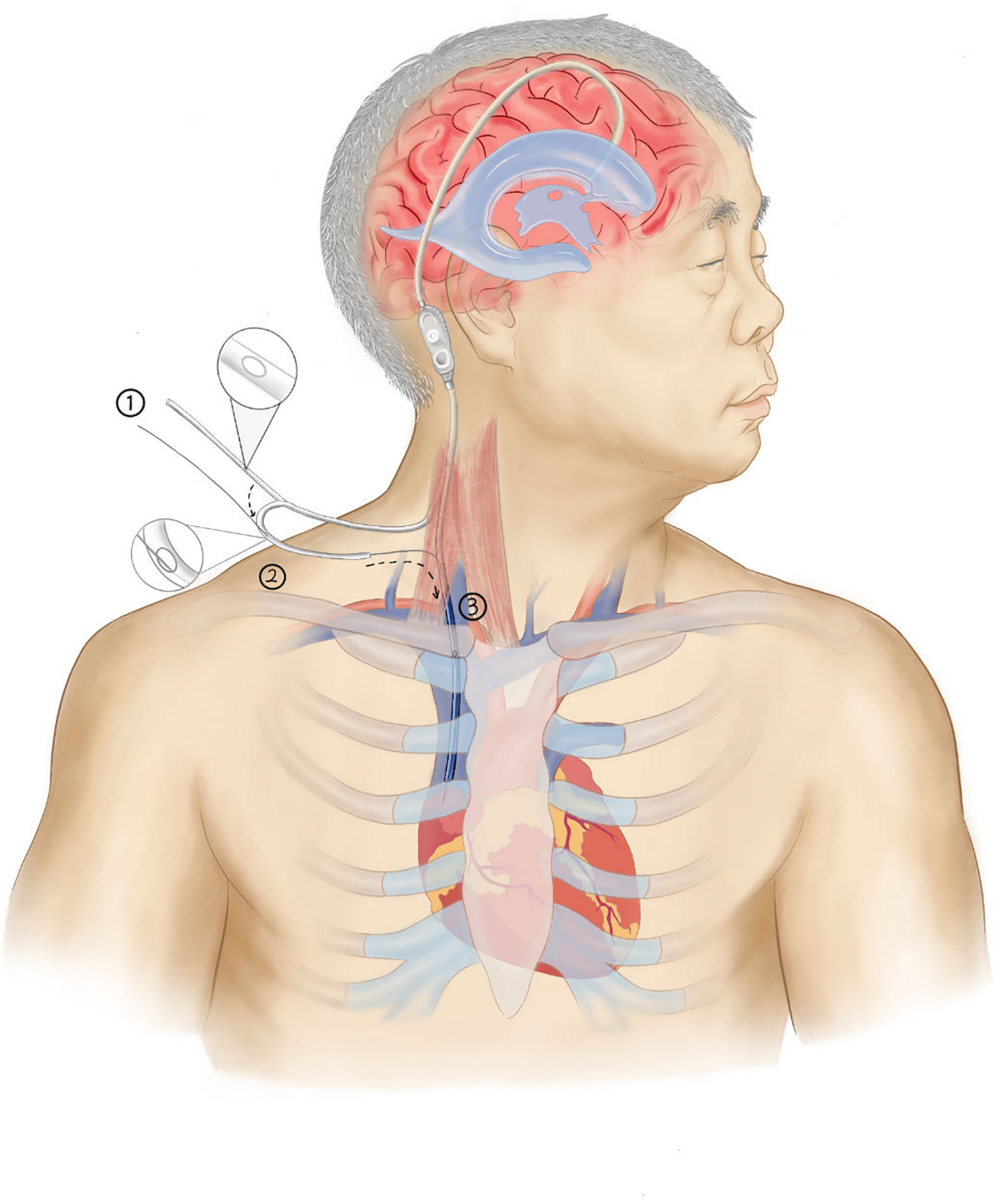
Schematic of the rapid exchange technique. (Step 1: After reserving a suitable catheter length (16 cm), cut an approximately 2 mm side hole at an appropriate location (6 cm from the end). Step 2: Pass the catheter tip over the guidewire and exit through the side hole. Step 3: Advance the catheter along the guidewire into the jugular vein and into the right atrium. Finally, remove the guidewire to complete the insertion.).

**Figure 4:**
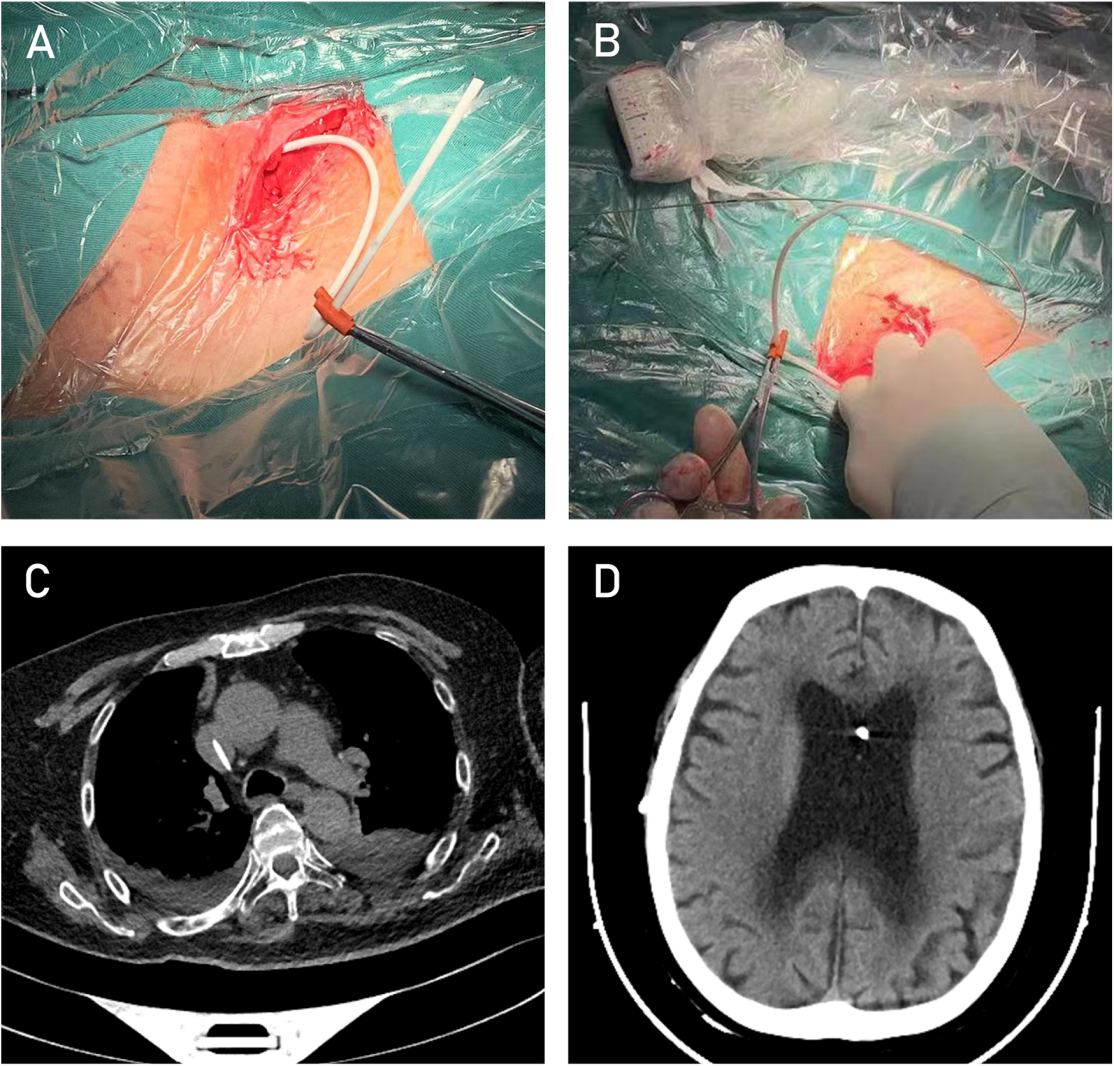
Intraoperative image after completion of Step 1. **(A)** Intraoperative image after completion of Step 2. **(B)** Post-VA shunt cardiac CT image showing the catheter in the target position. **(C)** Post-VA shunt follow-up CT image of the lateral ventricles **(D)**.

The detailed surgical steps are as follows:

(1) Using ultrasound to locate the internal jugular vein and the subcutaneous drainage tube, a small incision was made in the neck. The peritoneal end of the drainage catheter (The cerebrospinal fluid shunt device from Medtronic features a distal drainage catheter that is universally applicable for both cardiac and peritoneal settings, with an inner diameter of 1.3 mm and an outer diameter of 2.5 mm) was identified and pulled through this incision, with the excess length cut off, leaving approximately 16 cm of catheter remaining. (2) A hemostatic clamp with a protective sheath was temporarily applied to the drainage catheter to prevent excessive cerebrospinal fluid drainage. (3) A small side hole, approximately 2 mm in diameter, was created about 6 cm from the distal end of the drainage catheter (Figure 4A). This side hole is crucial for the “rapid exchange technique,” as it allows the guidewire to exit from the catheter. (4) The drainage catheter was soaked in heparinized saline, and heparinized saline was injected into the catheter through the side hole. (5) Under ultrasound guidance, a puncture kit was used to access the internal jugular vein through the neck incision. Using the Seldinger technique, the internal jugular vein was successfully punctured, and the tip of a “J”-shaped guidewire was inserted. The puncture needle was removed, and an 8F dilator was used to dilate the sternocleidomastoid muscle overlying the internal jugular vein, guided by the wire (Figure 4B). (6) The tail end of the guidewire was threaded through the drainage catheter and pulled out through the side hole. (7) While holding the guidewire's tail end, the drainage catheter was carefully advanced into the internal jugular vein. ECG monitoring revealed no significant arrhythmias, and ultrasound reconfirmed the guidewire's position within the vein. (8) The microguidewire was withdrawn, and the incision was sutured.This process is illustrated in the diagram (Figure 3).

After the surgery, the patient's hydrocephalus-related symptoms gradually improved. A head CT scan performed one week postoperatively showed a significant reduction in ventricular size (Figures 4C,D). At the 3-month follow-up, the patient's modified Rankin Scale (mRS) score was 1.

## Discussion and conclusions

The “coaxial technique” and “rapid exchange technique” are two commonly used methods in neurovascular interventional procedures. However, to our knowledge, this is the first report of using the “rapid exchange technique” in cerebrospinal fluid shunting procedures. The “rapid exchange technique” allows for quick, simple, and minimally invasive placement of the drainage catheter into the atrium or superior vena cava, with shorter operative time, less trauma, reduced risk of infection, and a lower likelihood of air embolism formation. It only requires a small incision in the neck, making it as straightforward as establishing venous access via internal jugular vein catheterisation. Once the technique is mastered, the procedure can even be performed under local anaesthesia without ultrasound guidance. A detailed comparison between the existing techniques and the present technique is shown in Table 1.

**Table 1:** “coaxial technique”: The guidewire passes through the catheter and remains aligned along the same axis. “rapid exchange technique”: The guidewire passes through the catheter and exits through the side opening of the catheter.

Study	Brief method	Exposing the shunt valve	Exposing the Jugular Vein	Duration of surgery
Pudenz (5)	Expose the jugular vein and insert the catheter into the vein under direct visualization until it reaches the atrium.	yes	yes	long
Oktay et al. (7)	Under ultrasound guidance, the jugular vein is percutaneously punctured, and the catheter is inserted using the “coaxial technique.” The catheter is then connected to the shunt valve in reverse through a subcutaneous tunnel.	yes	no	medium
Present case	Under ultrasound guidance, the jugular vein is percutaneously punctured, and the catheter is inserted into the atrium directly using the “rapid exchange technique,” without disconnecting the shunt valve.	no	no	short

However, several important considerations must be noted with this new surgical method: (1) It must be confirmed that the shunt obstruction is due to abdominal factors, with no issues present in the shunt catheter or valve itself. (2) There should be no signs of infection in the shunt system, ventricles, or abdomen, as this could lead to bloodstream infections. (3) The design of the side hole in the drainage catheter must be appropriate. It is crucial to ensure that once the distal end of the drainage catheter is fully inserted into the venous system, the side hole is positioned within the vein, not subcutaneously. If placed subcutaneously, it may lead to fluid accumulation under the skin of the neck, compression of nearby nerves, blood vessels, or organs, or even trigger an infection with serious consequences. If the side hole is too close to the catheter tip, it may interfere with the guidewire's function, leading to failure in catheter placement. However, designing the side hole placement is not difficult, and rough estimation can prevent significantly inappropriate positioning. Additionally, the side hole design helps maintain the patency of the shunt catheter. (4) Adequate dilation of the sternocleidomastoid muscle must be ensured, as the drainage catheter is relatively soft, and insufficient dilation can make it difficult for the catheter to pass through. (5) Whether anticoagulation is required after ventriculo-superior vena cava or atrial shunt placement remains unclear, with limited research available on the topic.

Although antiplatelet therapy may help prevent thrombosis in the drainage catheter, ensuring unobstructed drainage and reducing the risk of thromboembolic events carries an increased risk of bleeding (9). One study indicated that the postoperative administration of low-dose aspirin (3–5 mg/kg once daily) did not increase complication rates (10). Based rent literature, we chose to administer low-dose aspirin as antiplatelet therapy to minimize postoperative complications.

The rapid exchange technique is a quick, simple, and minimally invasive method for converting a VP shunt to a VA shunt, providing significant benefits for clinical practice.

## Data Availability

The original contributions presented in the study are included in the article/Supplementary Material, further inquiries can be directed to the corresponding author.
